# Supplementary material for: Rev Protein Diversity in HIV-1 Group M Clades
Source: Viruses. 2024 May 10;16(5):759. doi: 10.3390/v16050759 (PMC11125641; doi:10.3390/v16050759)
Supplement: Supplementary file 1 [file viruses-16-00759-s001.zip › Figure S1_Revised.pdf]

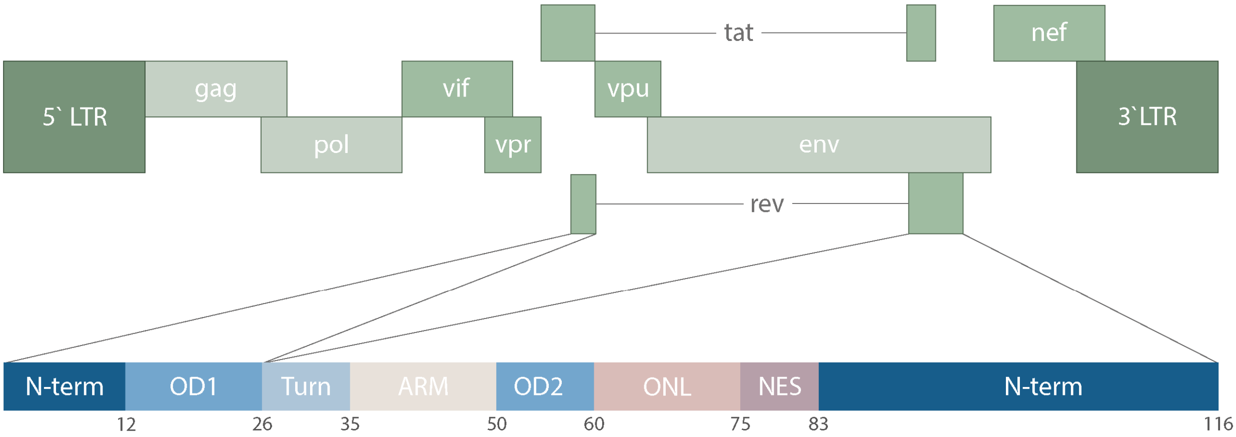

Figure S1. HIV-1 genome structure. *Rev* gene and domain organization of Rev protein (HXB2 numbering). Rev is encoded by two exons. N-term and OD domains are encoded by the first exon. The first exon is overlapped with the first exon of *tat*. In the 26th amino acid, there is a splice site, exon-exon junction. Turn, ARM, second OD, ONL, NES and C-term domains are encoded by the second exon. The second exon is overlapped with the second exon of *tat* and *env* gene. Schematic representation of Rev domain organization was adapted from [4] and used under a Creative Commons Attribution 4.0 International License (http://creativecommons.org/licenses/by/4.0/).
