# Supplementary material for: Rev Protein Diversity in HIV-1 Group M Clades
Source: Viruses. 2024 May 10;16(5):759. doi: 10.3390/v16050759 (PMC11125641; doi:10.3390/v16050759)
Supplement: Supplementary file 1 [file viruses-16-00759-s001.zip › Table S2_Revised.pdf]

Supplementary Table S2. The number of nearly full-genome HIV sequence from the Los Alamos HIV database for a rev gene analyzed in the present study (data available as of September 15, 2023).

HIV-1 form		No. sequences [a]	HIV-1 form	No. sequences	HIV-1 form	No. sequences
Sub(-sub)types	A1	251/245	33_01B	7/7	84_A1D	3/3
	A2	4/4	34_01B	3/3	85_BC	11/11
	A3	3/3	35_AD	22/22	87_cpx	3/3
	A4	3/3	36_cpx	3/3	88_BC	3/3
	A6	221/187	37_cpx	4/4	89_BF	9/9
	A7	3/3	38_BF	5/5	90_BF1	6/6
	A8	2/2	39_BF	3/3	91_cpx	10/10
	B	1726/1716	40_BF	4/4	92_C2U	7/7
	C	876/865	41_CD	3/3	93_cpx	3/3
	D	160/156	42_BF	17/17	94_cpx	6/6
	F1	74/74	43_02G	5/5	95_02B	3/3
	F2	14/14	44_BF	3/3	96_cpx	3/3
	G	91/91	45_cpx	5/5	97_01B	5/5
	H	10/10	46_BF	8/8	98_06B	1/1
	J	7/7	47_BF	3/3	99_BF	2/2
	K	2/2	48_01B	3/3	100_01C	3/3
CRFs	01_AE	627/613	49_cpx	4/4	101_01B	3/3
	02_AG	205/204	50_A1D	5/5	102_0107	2/2
	03_AB	4/4	51_01B	7/7	103_01B	10/10
	04_cpx	5/5	52_01B	3/3	104_0107	3/3
	05_DF	4/4	53_01B	4/4	105_0108	5/5
	06_cpx	23/17	54_01B	3/3	106_cpx	6/6
	07_BC	40/40	55_01B	9/9	107_01B	4/4
	08_BC	33/33	56_cpx	7/7	108_BC	5/5
	09_cpx	5/5	57_BC	7/7	109_0107	2/2
	10_CD	3/3	58_01B	6/6	110_BC	3/3
	11_cpx	24/23	59_01B	8/8	111_01C	8/8
	12_BF	14/14	60_BC	5/5	112_0107	4/4
	13_cpx	10/10	61_BC	3/3	113_01B	3/3
	14_BG	12/12	62_BC	3/3	114_01B	7/7
	15_01B	8/8	63_02A	26/26	115_01C	2/2
	16_A2D	4/4	64_BC	8/8	117_0107	3/3
	17_BF	7/6	65_cpx	6/5	118_BC	1/1
	18_cpx	7/7	66_BF1	8/8	119_0107	7/7
	19_cpx	5/5	67_01B	2/2	120_0107	3/3
	20_BG	5/5	68_01B	4/4	121_0107	4/4
	21_A2D	3/3	69_01B	7/7	122_BF1	4/4
	22_01A1	15/15	70_BF1	3/3	123_0107	3/3
	23_BG	2/2	71_BF1	14/14	124_cpx	2/2
	24_BG	4/4	72_BF1	6/6	125_0107	4/4
	25_cpx	6/6	73_BG	2/2	126_0755	4/4
	26_A5U	4/4	74_01B	3/3	129_56G	7/7
	27_cpx	4/3	75_BF1	3/3	130_A1B	1/1
	28_BF	5/5	76_01B	2/2	132_94B	5/5
	29_BF	6/6	77_cpx	4/4	133_A6B	8/8
	30_0206	3/3	79_0107	4/4	134_0107	6/6
	31_BC	3/3	80_0107	2/2	137_0107	9/9
	32_06A6	3/3	81_cpx	2/2	140_0107	5/5
					143_cpx	3/3

[a] Number sequences used in consensus calculation/ number nearly full-length HIV-1 genomes (See «Material and Methods»). HIV-1 subtypes/CRFs with more than available 10 sequences marked in red. CRF, circulating recombinant forms.
