# Supplementary material for: Rev Protein Diversity in HIV-1 Group M Clades
Source: Viruses. 2024 May 10;16(5):759. doi: 10.3390/v16050759 (PMC11125641; doi:10.3390/v16050759)
Supplement: Supplementary file 1 [file viruses-16-00759-s001.zip › Table S4_Revised.pdf]

Supplementary Table S4. Natural variations and average amino acid diversity at Rev individual domain within individual HIV-1 group M clades.

HIV- clade	Rev domain [a]							
	N-term	OD1	Turn	ARM	OD2	ONL	NES	C-term
	Mean changes per sequence (variable positions, %)							
A1	1.1 (83.3)	1.5 (78.6)	1.3 (66.7)	0.6 (80.0)	0.8 (100.0)	1.9 (100.0)	0.7 (75.0)	7.0 (100.0)
A6	0.8 (41.7)	1.0 (78.6)	0.6 (77.8)	1.2 (88.7)	1.1 (90.0)	1.7 (80.0)	0.3 (75.0)	2.8 (95.0)
B	0.8 (100.0)	2.0 (100.0)	1.1 (100.0)	0.5 (100.0)	2.8 (100.0)	2.8 (100.0)	0.3 (75.0)	4.6 (100.0)
C	0.7 (91.7)	0.9 (100.0)	0.8 (88.9)	0.7 (100.0)	1.0 (100.0)	2.3 (100.0)	0.6 (87.5)	3.9 (100.0)
D	0.9 (75.0)	1.7 (78.6)	0.6 (66.7)	0.2 (53.3)	0.9 (90.0)	0.9 (66.7)	0.6 (87.5)	4.1 (100.0)
F1	1.4 (50.0)	1.6 (71.4)	0.4 (55.5)	0.2 (40.0)	1.2 (80.0)	1.8 (80.0)	0.9 (62.5)	5.2 (90.9)
F2	0.5 (25.0)	0.6 (42.8)	0.6 (44.4)	0.4 (20.0)	0.8 (40.0)	2.4 (60.0)	0.3 (50.0)	5.9 (69.7)
G	1.1 (50.0)	1.6 (85.7)	0.7 (55.5)	0.6 (66.7)	1.1 (90.0)	2.6 (66.7)	0.9 (62.5)	6.8 (90.0)
H	1.8 (33.0)	2.2 (57.1)	1.0 (44.4)	0.5 (26.7)	1.5 (50.0)	2.4 (60.0)	0.2 (25.0)	3.9 (55.4)
01_AE	0.3 (100.0)	0.3 (100.0)	0.3 (100.0)	0.3 (100.0)	1.3 (90.0)	2.0 (93.3)	0.7 (87.5)	4.9 (97.5)
02_AG	0.9 (83.3)	1.5 (85.7)	0.8 (88.9)	0.6 (73.3)	1.5 (90.0)	2.1 (93.3)	0.9 (62.5)	7.0 (95.0)
06_cpx	0.9 (41.7)	1.0 (60.0)	1.3 (55.5)	0.6 (33.3)	0.8 (60.0)	1.9 (60.0)	0.7 (25.0)	2.7 (45.4)
07_BC	1.6 (58.3)	1.1 (50.0)	0.4 (55.5)	0.4 (33.3)	0.8 (70.0)	1.5 (53.3)	0.8 (50.0)	1.6 (75.0)
08_BC	0.2 (16.7)	0.4 (50.0)	0.6 (55.5)	1.3 (26.7)	0.5 (70.0)	0.9 (53.3)	0.2 (37.5)	1.4 (70.8)
11_cpx	0.8 (33.3)	2.1 (92.8)	1.9 (55.5)	0.9 (40.0)	0.8 (70.0)	2.1 (60.0)	0.7 (62.5)	4.6 (78.8)
12_BF	1.3 (33.3)	1.8 (42.8)	0.7 (33.3)	0.3 (26.7)	1.3 (50.0)	1.7 (53.3)	0.1 (25.0)	3.1 (45.4)
13_cpx	0.8 (25.0)	2.0 (50.0)	1.0 (33.3)	0.6 (13.3)	1.5 (60.0)	1.4 (40.0)	0.7 (37.5)	5.4 (47.5)
14_BG	0.4 (25.0)	0.9 (50.0)	0.2 (22.2)	0.5 (20.0)	0.6 (30.0)	1.4 (53.3)	0.1 (12.5)	1.6 (22.5)
22_01A1	1.1 (33.3)	1.7 (50.0)	1.5 (55.5)	0.7 (13.3)	0.5 (50.0)	2.7 (66.7)	0.9 (62.5)	3.7 (57.5)
35_A1D	0.4 (33.3)	1.5 (57.1)	0.9 (44.4)	0.6 (26.7)	0.1 (20.0)	1.3 (66.7)	0.5 (62.5)	2.8 (40.0)
42_BF1	0.3 (16.7)	0 (0)	0.1 (11.1)	0 (0)	0.3 (20.0)	0.2 (13.3)	0 (0)	0.3 (9.1)
63_02A6	0.7 (33.3)	1.0 (50.0)	0.2 (55.5)	0.2 (22.7)	1.2 (70.0)	1.3 (53.3)	0.8 (25.0)	2.1 (52.5)
71_BF1	0.7 (33.3)	2.0 (50.0)	0.9 (33.3)	0.4 (20.0)	2.3 (80.0)	2.1 (60.0)	0.2 (37.5)	3.3 (48.5)
85_BC	0.7 (33.3)	1.1 (35.7)	0.5 (44.4)	0.7 (33.3)	0.9 (50.0)	0.4 (26.7)	0.2 (25.0)	0.7 (25.0)
91_cpx	0.3 (8.3)	0.7 (42.8)	0.2 (22.2)	0.4 (13.3)	0.8 (50.0)	0.8 (26.7)	0.1 (12.5)	0.5 (12.1)
103_01B	0.1 (8.3)	1.3 (50.0)	1.4 (44.4)	0.5 (13.3)	1.4 (50.0)	2.4 (46.07)	0.1 (12.5)	2.0 (27.5)
	Median [IQR] average amino acid diversity (10^{-2} substitutions/site)							

A1	16.9 (11.6)	14.7 (15.7)	28.9 (31.7)	6.6 (13.5)	9.6 (11.3)	23.9 (18.5)	15.9 (18.1)	26.8 (16.3)
A6	9.1 (18.80)	13.9 (9.7)	13.9 (28.4)	13.5 (10.1)	19.6 (16.8)	22.7 (18.5)	0 (15.9)	9.6 (8.4)
B	8.4 (9.5)	25.2 (20.6)	26.3 (18.8)	6.8 (7.3)	55.0 (38.4)	36.5 (24.8)	0 (15.5)	23.6 (15.0)
C	8.4 (17.4)	13.9 (10.4)	16.6 (36.8)	6.6 (13.5)	19.4 (19.0)	28.6 (25.5)	14.6 (30.6)	28.0 (19.3)
D	8.8 (10.1)	22.4 (13.5)	12.8 (15.7)	0 (0)	11.9 (14.3)	7.8 (11.0)	15.6 (16.5)	20.6 (12.4)
F1	16.7 (8.3)	14.3 (7.1)	0 (11.1)	0 (6. 7)	20.0 (20.0)	20.0 (13.3)	12.5 (25.0)	24.2 (12.1)
F2	8.3 (8.3)	7.1 (14.3)	11.1 (11.1)	6.7 (6.7)	10.0 (10.0)	26.7 (13.3)	12.5 (12.5)	27.3 (9.1)
G	16.7 (8.3)	15.4 (7.1)	11.1 (22.2)	6.7 (6.7)	20.0 (20.0)	26.7 (13.3)	12.5 (12.5)	23.1 (9.3)
H	25.0 (8.3)	21.4 (14.3)	22.2 (22.2)	6.7 (0)	20.0 (10.0)	26.7 (13.3)	0 (12.5)	19.5 (9.1)
01_AE	0 (8.3)	14.0 (15.0)	25.1 (20.9)	6.7 (6.9)	20.3 (23.5)	24.8 (20.6)	15.9 (16.6)	16.7 (10.5)
02_AG	10.4 (11.1)	15.2 (15.9)	15.0 (18.9)	6.6 (13.5)	21.8 (24.8)	25.6 (22.9)	15.7 (18.5)	25.0 (13.6)
06_cpx	14.6 (10.9)	9.6 (23.2)	29.2 (30.3)	6.6 (13.6)	9.6 (23.2)	25.1 (21.0)	15.3 (34.3)	16.2 (10.9)
07_BC	18.7 (20.0)	14.1 (14.8)	13.4 (13.8)	6.6 (7.7)	11.0 (19.9)	15.7 (17.0)	14.6 (35.6)	12.7 (9.3)
08_BC	0 (8.6)	0 (6.9)	13.5 (28.3)	13.5 (11.2)	9.1 (19.3)	15.3 (10.2)	0 (15.9)	8.7 (11.3)
11_cpx	8.5 (8.9)	22.9 (23.3)	44.3 (28.5)	13.3 (7.8)	9.5 (21.0)	27.2 (17.1)	17.0 (36.6)	23.8 (15.0)
12_BF	18.2 (13.8)	22.7 (9.9)	14.0 (13.5)	6.6 (6.7)	20.1 (22.7)	27.9 (18.2)	0 (0)	17.1 (12.8)
13_cpx	8.9 (9.9)	23.4 (12.2)	27.5 (16.6)	6.6 (6.8)	19.9 (23.2)	17.7 (18.6)	15.9 (18.8)	19.9 (10.2)
14_BG	8.1 (9.7)	7.2 (9.3)	0 (13.5)	6.6 (6.7)	8.9 (19.2)	14.0 (15.5)	0 (0)	7.4 (3.4)
22_01A1	9.3 (10.4)	18.4 (15.8)	45.1 (23.8)	6.7 (1.2)	9.6 (19.8)	37.4 (25.1)	33.4 (23.1)	17.1 (8.9)
35_A1D	0 (9.6)	15.1 (17.6)	13.8 (15.6)	6.5 (6.5)	0 (8.8)	15.8 (18.4)	15.8 (32.1)	10.4 (5.6)
42_BF1	8.1 (8.2)	0 (0)	0 (0)	0 (0)	0 (11.2)	0 (7.6)	0 (0)	0 (3.1)
63_02A6	8.9 (20.0)	8.0 (21.9)	0 (13.5)	0 (0)	21.0 (25.0)	15.8 (20.0)	13.6 (28.9)	7.4 (10.5)
71_BF1	8.4 (10.2)	25.5 (16.6)	25.7 (17.7)	6.7 (6.8)	46.2 (28.3)	25.7 (20.8)	0 (15.5)	17.7 (9.4)
85_BC	8.2 (18.3)	14.0 (14.0)	13.4 (13.7)	6.8 (13.8)	18.8 (30.0)	0 (15.9)	0 (14.4)	4.1 (4.3)
91_cpx	0 (8.8)	7.4 (8.4)	0 (0)	6.6 (6.6)	9.5 (11.0)	8.2 (9.0)	0 (0)	3.3 (3.5)
103_01B	0 (0)	14.8 (7.7)	28.6 (24.4)	6.6 (6.7)	21.3 (21.2)	34.2 (23.1)	0 (0)	8.1 (8.6)

[a] Annotated protein domains are indicated as colored rectangles: N-term, N-terminal region; OD, Oligomerization domain; ARM, Arginine-Rich Motif; NES, Nuclear Export Signal; C-term, C-terminal region; ONL, OD-NES Linker. IQR, interquartile range (25% - 75%, Q1-Q3).
