# Supplementary material for: Rev Protein Diversity in HIV-1 Group M Clades
Source: Viruses. 2024 May 10;16(5):759. doi: 10.3390/v16050759 (PMC11125641; doi:10.3390/v16050759)
Supplement: Supplementary file 1 [file viruses-16-00759-s001.zip › Table S6_Revised.pdf]

Supplementary Table S6. Range of conservation of individual Rev domain across different HIV-1 group M clades.

Clade [a]	Conservation (%)										
	64.5 – 72.1	72.2 – 74.9	75.0-77.9	78.0-80.9	81.0-83.9	84.0-86.9	87.0-89.9	90.0-92.9	93.0-95.9	96.0-98.9	99.0-100
Group M	C-term			Turn	OD2, ONL	NES	N-term, OD1		ARM		
A1					C-term	Turn	OD1	N-term, OD2, NES	ARM		
A6							OD2, ONL	N-term, OD1, Turn, ARM, C-term	NES		
B		OD2			ONL	OD1, C-term	Turn		N-term	ARM, NES	
C				C-term		ONL	OD2	Turn, NES	N-term, OD1, ARM		
D							OD1, C-term	N-term, OD2, NES, Turn	ONL	ARM	
F1						C-term	N-term, OD1, OD2, ONL, NES		Turn	ARM	
F2					ONL, C-term			OD2	N-term, Turn, NES, OD1	ARM	
G					ONL, C-term		OD1, OD2, NES	N-term, Turn	ARM		
H						N-term, OD1, OD2, ONL	Turn, C-term			ARM, NES	
01_AE						Turn, ONL	OD2, C-term	OD1, ARM, NES		N-term	
02_AG					C-term	OD2, ONL	OD1, NES	N-term, Turn	ARM		
06_cpx						Turn	ONL	N-term, OD1, OD2, NES, C-term	ARM		
07_BC						N-term	C-term	OD1, OD2, ONL, NES	Turn	ARM	
08_BC								ARM, C-term	Turn, OD2, ONL	N-term, OD1, NES	
11_cpx				Turn		OD1, ONL, C-term		OD2, NES	N-term		
12_BF							N-term, OD1, OD2, ONL	Turn, C-term		ARM, NES	
13_cpx						OD1, OD2, C-term	Turn	ONL, NES	N-term	ARM	
14_BG								OD1, ONL	OD2, C-term	N-term, Turn, ARM, NES	
22_01A1					Turn, ONL		OD1, NES	N-term, C-term	ARM, OD2		
35_A1D						OD1	Turn	ONL, NES, C-term	N-term, ARM	OD2	

42_BF1						OD2	N-term, Turn, ONL, C-term	OD1, ARM, NES
63_02A6				OD2, ONL	N-term, OD1, NES	Turn, C-term	ARM	
71_BF1	OD2		OD1, ONL	Turn, C-term		N-term	ARM, NES	
85_BC					OD1,OD2	N-term, Turn, ARM	ONL, NES, C-term	
91_cpx					OD2	OD1, ONL	N-term, Turn, ARM,NES, C-term	
103_01B		Turn	OD2, ONL		OD1	C-term	ARM, NES	N-term

Annotated protein domains: N-term, N-terminal region; OD, Oligomerization domain; ARM, Arginine-Rich Motif; NES, Nuclear Export Signal; C-term, C-terminal region; ONL, OD-NES Linker.
